# Layer-by-Layer Deposition of Hollow TiO_2_ Spheres with Enhanced Photoelectric Conversion Efficiency for Dye-Sensitized Solar Cell Applications

**DOI:** 10.3390/nano14221782

**Published:** 2024-11-06

**Authors:** Rama Krishna Chava, Yeon-Tae Yu, Misook Kang

**Affiliations:** 1 Department of Chemistry, College of Natural Sciences, Yeungnam University, 280 Daehak-Ro, Gyeongsan 38541, Republic of Korea; drcrkphysics@hotmail.com; 2 Division of Advanced Materials Engineering, Jeonbuk National University, 567 Baekje-daero, Deokjin-gu, Jeonju 54896, Republic of Korea

**Keywords:** TiO_2_ hollow microspheres, light-scattering layer, electrophoretic deposition, bi- and tri-layers, photoanodes, dye sensitized solar cells, photoconversion efficiency

## Abstract

Fabricating photoanodes with a strong light-scattering effect can improve the photoconversion efficiency of dye-sensitized solar cells (DSSCs). In this work, a facile microwave hydrothermal process was developed to prepare Au@TiO_2_ core–shell nanostructures, and then the Au core was removed by etching, resulting in hollow TiO_2_. Morphological characterizations such as field emission scanning and transmission electron microscopy measurements have been used for the successful formation of core–shell and hollow TiO_2_ nanostructures. Next, we attempted to deposit the different-sized hollow TiO_2_-based microspheres simultaneously on the surface of small-sized TiO_2_ nanoparticles-based compact film as light-scattering layers via electrophoretic deposition. The deposited hollow TiO_2_ microspheres constitute bi- and tri-layers that not only improve the light-harvesting properties but also speed up the photogenerated charge transfer. Compared to commercial TiO_2_ compact film (4.75%), the resulting bi-layer and tri-layered films-based DSSCs displayed power conversion efficiencies of 6.33% and 8.08%, respectively. It is revealed that the deposited bi- and tri-layered films can enhance the light absorption ability via multiple photon reflection. This work validates a novel and controllable strategy to develop light-scattering layers with increased light-harvesting properties for highly efficient dye-sensitized solar cells.

## 1. Introduction

Dye-sensitized solar cells (DSSCs) have received extensive interest as one of the potential replacements for conventional silicon-based solar cells and intermediate-band solar cells due to their low cost, high efficiency and ease of fabrication [1,2,3,4,5,6]. In order to fabricate the efficient DSSC, the photoanode must exhibit the following characteristics such as greater light harvesting, higher surface area to accommodate numerous dye molecules, superior electron transportation and collection, suitable porosity to diffuse electrolyte inside of the film, etc. [7,8]. In most cases, nanosized semiconductors such as TiO_2_ nanoparticles smaller than ≈50 nm have been used as mesoporous layers in photoanodes because of their higher surface-to-volume ratio. However, this mesoporous layer with a particle size of less than 50 nm cannot sufficiently reflect or scatter light, and as a result, lower light harvesting is observed [9,10]. Therefore, to avoid these issues, researchers have introduced light-scattering materials with submicron-sized large particles in the photoanodes, which are usually termed bi-layered films [11,12]. For example, large popcorn-like nanostructures with a diameter of around 300 nm were applied as a light-scattering layer (LSL), and as a result, an enhanced photoconversion efficiency (PCE) of 7.56% was achieved [13]. A new type of TiO_2_ hollow spheres with a shell-in-shell type morphology was introduced by Wu et al., where the Ti-precursor TiF_4_ acts as a controlling agent in adjusting the shell thickness and hollow structures. The greater light-scattering properties of shell-in-shell TiO_2_ make it as an excellent choice in DSSCs. The resultant films displayed an overall PCE of 9.10% which is 20% higher than the pristine P-25 films under AM-1.5G one sunlight intensity [14]. Next, Chu et al. introduced mesoporous TiO_2_ microspheres with a size of 400 nm in the photoelectrodes in which TiO_2_ microspheres with interconnected nanoparticles that offer higher specific surface area, higher light-scattering property, and long-range electron connectivity exhibited an enhanced PCE of 7.13% [15]. Likewise, nanopetals consisting of microflower-like TiO_2_ with a high surface area were reported by Shih et al. [16] and applied as LSL in the photoanodes of DSSCs. The obtained bi-film layer provides a large surface area for dye adsorption and direct pathways for electron transportation. The effective light scattering resulting from the large particle size of 2–3 μm increases the light-harvesting capacity and, as a result, an efficiency of 5.30% is observed with its counterpart (3.90%). In another way, light scattering can also be achieved by making a mixed structure in which large particles as light scatterers are embedded into the small-sized nanoparticle film. For instance, mesoporous ZnO hollow microspheres loaded into the SnO_2_-based DSSCs and TiO_2_ hollow spheres-embedded TiO_2_ nanocrystalline photoanodes were reported [17,18], in which the better light-harvesting properties, high surface area of ZnO hollow spheres, excellent infiltration of electrolyte into the photoanode film and better interfacial charge transport improved the J_sc_ of the DSSCs with a fill factor of 65%. Based on these results, the fabrication of bi-layered photoanodes for DSSC applications are essential in realizing higher photoconversion efficiencies.

Generally, light-scattering layers on the compact TiO_2_ layer are obtained via the doctor blade method, screen printing, spin coating etc. The coating of LSL and further calcination process results in a loose particle-packing network on compact film and hence offers electron path resistance, decreasing the photoconversion efficiency. Another drawback of these methods is the precise control of the thickness of the light-scattering layer. Alternatively, electrophoretic deposition (EPD) has emerged as one of the well-fabrication methods to make photoanodes for DSSC applications. Previously, EPD was a well-known approach for depositing materials such as metallic, insulating, nanoparticles and semiconductors on conducting substrates [19]. EPD is a technique based on the motion and grafting of charged particles under an applied electric field, and it has several advantages such as low-cost and short-time deposition, simple apparatus and the ability to form uniform particle layers with controlled thickness [20,21]. Therefore, several researchers attempted to make TiO_2_-based films via the EPD method and successfully achieved neat, uniform, and good compact layers on the substrates, which were further tested in DSSC applications [22,23,24]. Recently, hollow nanoparticles with a diameter above 100 nm have been considered as promising light-scattering materials for DSSC applications. These hollow-type particles have a beneficial configuration of multiple reflections and redox reactions within the electrolyte. To date, various hollow nanoparticles have been synthesized, such as shell-in-shell TiO_2_ hollow spheres [14], multi-shell porous TiO_2_ [25], TiO_2_-coated multilayered SnO_2_ hollow microspheres [26], SnO_2_ hollow microspheres [27], multi-shelled ZnO hollow microspheres [28], etc. These hollow nanostructures with higher specific surface area offered excellent light-harvesting properties and displayed excellent photovoltaic conversion efficiency.

Benefitting from the advantages of the hollow structured morphology and EPD process, herein, we attempted to fabricate a novel tri-layered film that consists of TiO_2_ hollow particles with different sizes. In this work, we utilized a microwave hydrothermal synthesis method for the preparation of hollow TiO_2_ particles which were derived from the Au selective etching of Au@TiO_2_ core–shell particles. The hollow TiO_2_ nanoparticles (HTNPs) used in this study are composed of aggregated spherical shapes with hollow spaces left behind after the etching of Au NPs. The obtained HTNPs were expected to offer a higher specific surface area, multi-reflection abilities, and greater light-harvesting capacity. The resultant films obtained via the EPD process were used as photoanodes in DSSCs and displayed an overall conversion efficiency of 8.08% under one sunlight intensity.

## 2. Materials and Methods

### 2.1. Synthesis of Hollow TiO_2_ NPs (HTNPs)

In this work, hollow TiO_2_ nanoparticles were synthesized in two steps according to our previous report [29]. Initially, Au@TiO_2_ core–shell NPs with different core sizes were synthesized by the microwave method. Briefly, the precursors 1 mL of HAuCl_4_ (0.01 M), 2 mL of trisodium citrate (0.01 M) and 1.2 mL of 0.01 M ascorbic acid were sequentially added in a 20 mL glass vial and kept stirring for 15 min. After stirring, 3 mL of 0.04 M TiF_4_ solution was added dropwise under constant stirring. Next, the resultant solution mixture was diluted to 30 mL with double-distilled (DI) water, and then the finally obtained solution was transferred to a Teflon liner and sealed. Then, the sealed Teflon liner was treated in a commercial microwave reactor for one hour at 180 °C. After the reactor vessel was cooled down naturally, the Au-TiO_2_ core–shell NPs precipitate was washed and centrifuged several times with distilled water and then dispersed in 20 mL DI water for a further Au etching process. In the next step, 5 mL of 0.01 M KCN solution was freshly prepared, added to the Au-TiO_2_ core–shell NP solution, and stirred for a few minutes. The pH of the resultant solution was changed to 10.5 and continuously stirred for another 3 h. The resultant residue was washed and centrifuged five times with DI water and dispersed in 15 mL ethanol. The obtained TiO_2_ NPs with a hollow interior of 20–30 nm after Au removal are denoted as HTNPs-1. Similarly, the other hollow TiO_2_ NPs with an interior space of around 70–80 nm (after Au NP etching) were also synthesized under identical conditions by simply changing the precursor concentrations (4.5 mL of 0.01 M HAuCl_4_; 4.5 mL of 0.01 M trisodium citrate; 6 mL of 0.01 M ascorbic acid and 3 mL of 0.04 M TiF_4_) and denoted as HTNPs-2.

### 2.2. Electrophoretic Deposition of HTiO_2_ NPs on TiO_2_ Compact Layer

The above-prepared 15 mL H-TiO_2_ NPs solution was diluted to 30 mL with ethanol, and the solution was sonicated for 10 min and then stirred for overnight to avoid agglomeration. The pH of the solution was changed to 13 using 1 M NaOH solution. The resultant colloidal solution was used for electrophoretic deposition. The fluorine-doped SnO_2_ (FTO) glass substrates were washed with DI water, acetone and ethanol sequentially by sonication and dried in an oven for 1 h. The first layer of commercial TiO_2_ NPs (25 nm in size)-based film with an active area of 0.1 cm^2^ was coated on the surface of the FTO substrate by the screen-printing method, and the average thickness of the film was about 8.5 μm. Then, the films were calcined at 450 °C for one hour and further used as electrodes in the electrophoretic deposition process. After several experiments, electrophoretic deposition was optimized and carried out at 50 V. During the deposition process, the FTO substrate with a TiO_2_ compact layer was used as the working electrode and the Pt plate was used as the counter electrode. The working electrode and counter electrode were arranged in a way that faced each other with a fixed distance of 1 cm. After the deposition, finally, the films were rinsed in ethanol and dried at 60 °C for several hours.

### 2.3. Photoanodes and DSSC Device Fabrication

At first, the above prepared bi-layered and tri-layered TiO_2_ films were calcined at 400 °C for one hour. After being cooled down to room temperature, the resultant films were sensitized with N719 Ruthenium dye ethanol solution (0.3 mM) overnight. After that, the films were washed with ethanol several times and then dried at room temperature for one hour and termed photoanodes. The platinum-based counter electrode (obtained by Pt sputtering on a predrilled FTO glass substrate) served as a photocathode/counter electrode. Next, the dye sensitized photoanode was assembled with a counter electrode using a thermal–plastic Surlyn spacer of 25 μm size as the spacer to design sandwich-type solar cells. A liquid electrolyte, which contained 0.6 M 1-butyl-3-methylimidazoliumiodide (BMII), 0.1 M guanidiniumthiocyanate (GuSCN), 0.03 M I_2_, and 0.5 M 4-tertbutylpyridine in acetonitrile–valeronitrile (85:15, vol%) was injected into the gap between two electrodes via a predrilled hole on the counter electrode side. The injection holes were sealed with transparent tape. Finally, the active area of dye-sensitized TiO_2_ film is around 0.1 cm^2^. For comparison, a DSSC device containing conventional screen-printed with commercial TiO_2_ paste as a single-layer film was also designed and compared with the efficiencies of EPD films-based DSSCs.

### 2.4. Characterization of Samples

The TEM images of the prepared Au@TiO_2_ and H-TiO_2_ NPs were recorded on a Hitachi H-7600 transmission electron microscope (TEM) with an accelerating voltage of 200 kV. After drying, the deposition patterns and cross-sectional thickness of the TiO_2_ films were studied using a Field Emission Scanning Electron Microscope (FESEM, Hitachi SU-70, operating voltage of 10 kV and current of 15 mA). The photocurrent–voltage (I-V) characteristics of fabricated DSSCs were measured on a McScience K201 Solar Simulator Lab100 under illumination with a power density of AM 1.5 (100 mW/cm^2^). External quantum efficiency (IPCE) recordings were taken from a McScience K3100 spectral IPCE measurement system.

## 3. Results and Discussion

**Figure 1:**
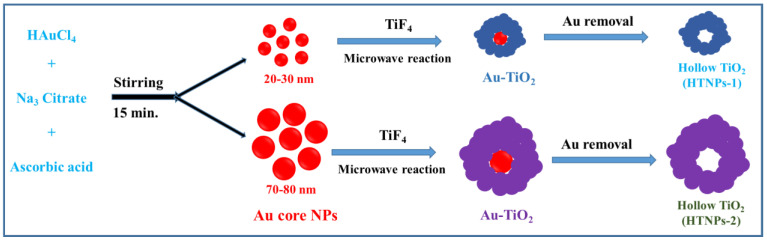
Illustration for the synthesis of Au-TiO_2_ core–shell and hollow TiO_2_ NPs.

The synthesis of hollow TiO_2_ NPs was carried out in two steps by microwave hydrothermal reaction and a subsequent Au selective etching process (Figure 1). Initially, Au NPs were prepared from chloroauric acid as the Au precursor, trisodium citrate as the stabilizing agent and ascorbic acid as the reducing agent. In the next step, adding TiF_4_ solution and subsequent microwave hydrothermal treatment resulted in the TiO_2_ shell formation on the surface of Au NPs, ensuring Au-TiO_2_ core–shell NPs growth. The added precursor TiF_4_ hydrolyzes into TiO_2_ by forming HF, which directs the growth of the TiO_2_ crystals and aggregated onto the surface of Au NPs as flower-shaped structures, and their corresponding reactions are as follows [30].

TiF_4_ + 4H_2_O → Ti(OH)_4_ + 4HF (1)

Ti(OH)_4_ → TiO_2_ + 2H_2_O (2)

**Figure 2:**
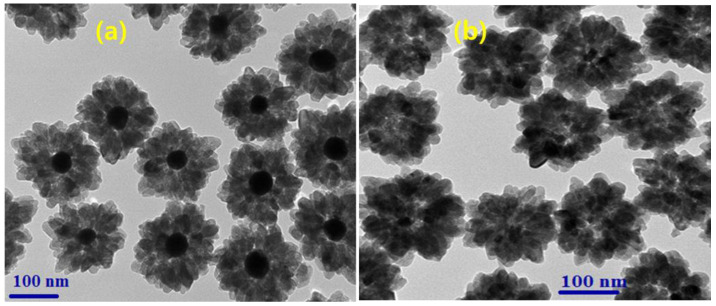
TEM images of Au-TiO_2_ core–shell and hollow TiO_2_ NPs (HTNPs-1).

The morphology of the final products was tested, and Figure 2 shows the TEM images of the prepared samples. As seen in Figure 2a, the morphology of the products obtained from the microwave hydrothermal reaction represents the core–shell NPs with a flower-shaped shell surrounding the Au-core NPs. The size of Au NPs is 20–30 nm, whereas the shell is about 100 nm. Next, Figure 2b represents the hollow TiO_2_ NPs in which the Au core was completely removed via a KCN etching process. The space/interior left behind in the removal of the Au NPs constitutes a hollow-type morphology, which is beneficial for increasing the light-scattering properties. During the etching process, the pH of the solution was maintained at around 10.5, which is crucial for obtaining the hollow-type morphology without damaging the spherical-shaped morphology.

### 3.1. Electrophoretic Deposition, Morphological Studies of HTNPs-Based Films

**Figure 3:**
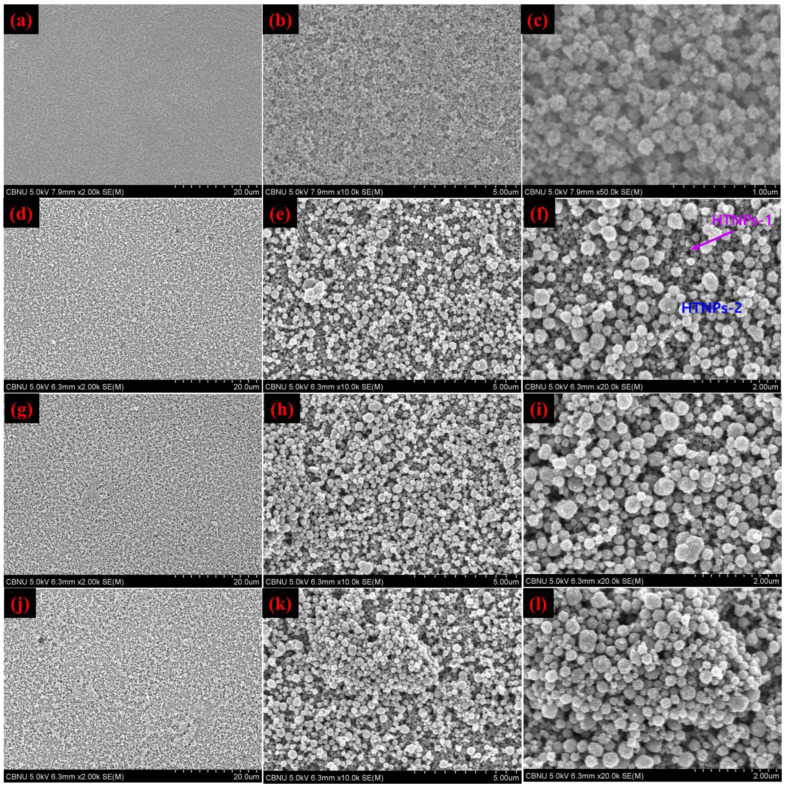
FE-SEM images of electrophoretically deposited HTNP films: (**a**–**c**) EPD of HTNPs-1 on TiO_2_ compact layer for 10 min that formed a bi-layer film, EPD of HTNPs-2 on bi-layered films for 5 min (**d**–**f**), 10 min (**g**–**i**) and 15 min (**j**–**l**) that resulted in tri-layered films.

EPD is a novel method used to accumulate the NPs and rapidly deposits them on the substrates as dense films, whose thickness could be tuned by changing the deposition time and applied potential. Among the deposition techniques, EPD alone offers the enhanced deposition rates and harvests films with low surface roughness and long-range consistency in morphology and thickness [31]. In this work, EPD was performed in an ethanol suspension containing HTNPs. Before starting the EPD experiments, a TiO_2_ compact layer was deposited on the FTO substrate by the traditional screen-printing method, which was followed by calcination at higher temperature, resulting in a compact layer of thickness of about 8.5 μm and an active area of 0.1 cm^2^. For the EPD of HTNPs, the FTO substrate with a compact layer was immersed in the ethanol solution of HTNPs, and a DC voltage of 50 V was applied for 10 min. The parameters such as time and voltage are optimized after several experiments. After the EPD experiments, the films obtained were analyzed by taking the FE-SEM images in each step, and they are provided in Figure 3. As seen in Figure 3a–c and Figure S1, the FE-SEM images clearly show the uniform deposition of HTNPs-1 on the surface TiO_2_ compact layer, resulting in a bi-layered film. From low to high-magnified images in Figure 3a–c, it is confirmed that the EPD could form a thick and uniform porous layer which is highly beneficial in infiltrating the dye and electrolyte molecules deep into the film. In this work, our goal is to make a tri-layered film that consists of a TiO_2_ compact layer (15–20 nm sized TiO_2_) and two light-scattering layers of HTNPs-1-film followed by HTNPs-2 based film. Therefore, the above obtained bi-layered film was used for further EPD experiments for 5, 10 and 15 min under the external electric field strength of 50 V. The relevant films were also characterized, and their corresponding FE-SEM images are provided in Figure 3d–l. According to Figure 3d–f (EPD of 5 min), the deposition of HTNPs-2 also occurred on the surface of the bi-layered film, and upon increasing the deposition time to 10 (Figure 3g–i) and 15 min (Figure 3j–l), the concentration of the deposited HTNPs-2 was also increased on the HTNPs-1 film, resulting a tri-layered film. To avoid the cracking or peeling of the films, the deposition time was limited to 15 min. Based on the surface morphology of the films, it is confirmed that EPD could form crack-free films with a controllable thickness.

### 3.2. DSSCs Performance of HTNPs-Based Films

**Figure 4:**
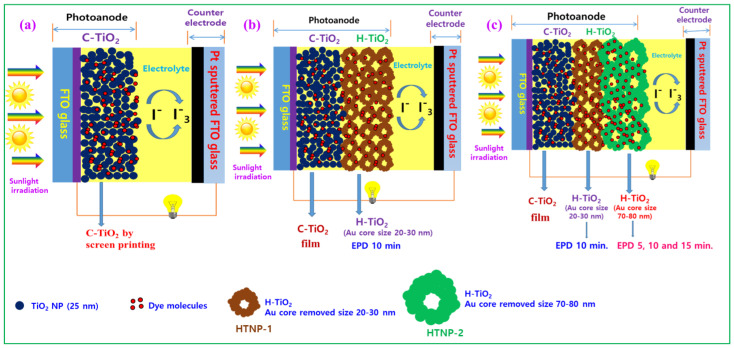
Fabrication details of DSSCs containing different photoanodes derived from EPD experiments. (**a**) DSSC device prepared from the commercial TiO_2_ NPs film via screen-printing method, (**b**) DSSC device prepared from the electrophoretic deposition of HTNP-1 on commercial TiO_2_ NPs film (bi-layer), (**c**) DSSC device prepared from the electrophoretic deposition of HTNP-1 and HTNP-2 on commercial TiO_2_ NPs film.

**Figure 5:**
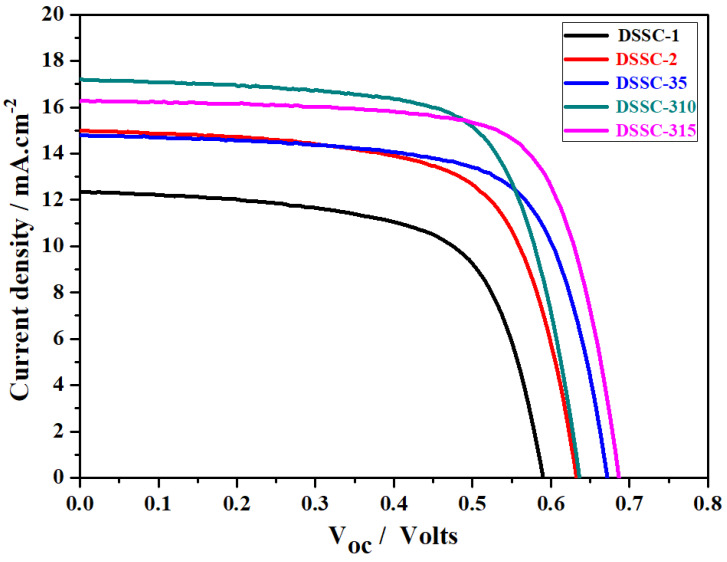
J-V plots of DSSCs made from TiO_2_ compact layer and electrophoretically deposited HTNPs-based films.

**Figure 6:**
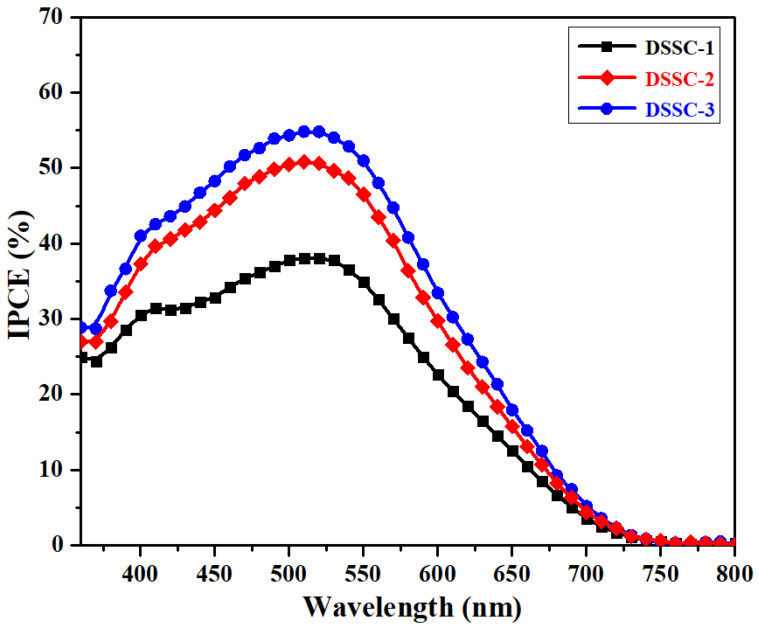
IPCE spectra of the DSSC devices prepared from C-TiO2 and EPD of HTNPs-1 and HTNPs-2 films.

After making the HTNPs-based bi- and tri-layered films, DSSCs were developed, and we tested the photovoltaic performance of the cells under 1 sun radiation. The fabrication of DSSCs based on various photoanodes that contain different light-scattering layers are presented in Figure 4. The J-V characteristics of the DSSCs made from the photoanodes of the TiO_2_ compact layer and EPD of the light-scattering layers of HTNPs-1 and HTNPs-2 are shown in Figure 5, and their corresponding parameters are provided in Table 1. For convenience, the DSSCs designed from a single compact layer of TiO_2_ are denoted as DSSC-1 whereas those designed with the light-scattering layer of the HTNPs-1 film (which were obtained via the EPD of HTNPs-2 for 10 min and resulted in a bi-layered film) are denoted as DSSC-2. Next, the DSSCs obtained from the EPD of HTNPs-2 on bi-layered films are noted as DSSC-35, DSSC-310 and DSSC-315, representing the EPD of HTNPs-2 at the deposition times of 5, 10 and 15 min, respectively. As seen in Figure 5, the photovoltaic devices obtained from the TiO_2_ compact layer (DSSC-1) exhibited a V_oc_ ≈ 0.59 V, J_sc_ ≈ 12.34 mA/cm^2^ and a fill factor of 65.27%, affording a photoconversion efficiency of 4.75%. Meanwhile, bi-layered photoanode-containing devices (DSSC-2) showed an increased performance viz. V_oc_ ≈ 0.63 V, J_sc_ ≈ 15.00 mA/cm^2^ and a fill factor of 66.77% with a PCE of 6.33%. Next, the EPD of HTNPs-2 on the top of the bi-layered film and subsequent fabricated devices resulting in tri-layered films showed a significant increase in photoconversion efficiency. According to Figure 5 and Table 1, as the deposition time increases, the PCE also increases and reaches a maximum PCE of 8.08%. The enhancement in PCE from 6.90 to 8.08% signifies the importance of the light-scattering layer of HTNPs-2 for photovoltaic applications. The improvement in the short-circuit voltage (Voc) and current density (Jsc) led to increases in the device efficiency of DSSC-2 and DSSC-3. The enhancement in the PCE of DSSC-2 and DSSC-3 is due to the improved light-harvesting capacity of photoanodes with the light-scattering layers of HTNPs-1 and HTNPs-2 and decreased electron–hole recombination of photoinduced electrons at the semiconductor/electrolyte interface. Therefore, the efficient light-harvesting properties are the main reason for the increase in the light reflection/scattering properties of the HTNPs-based photoanodes, permitting the trapping of incident light by scattering in the hollow cavities of HTNPs and amplifying the optical path of the incident light, which causes the interaction with the incident photons and adsorbed dye molecules [32,33,34]. As a result, the current density (Jsc) of photoanodes was greatly improved from 12.34 to 16.26 mA/cm^2^. A noticeable increment in the FF values represents the strong contact between the films and substrates, implying the EPD process is a convenient approach in fabricating the films for photovoltaic applications [35]. The light-harvesting capacities of the prepared films were characterized by taking the incident photon to conversion efficiency (IPCE), since the IPCE spectra reflect the light response behavior of photoanodes, which is directly connected to the photocurrent density. Therefore, as seen in Figure 6, the DSSC devices with the light scattering of HTNPs-2 possessed higher IPCE intensity than those of the TiO_2_ compact layer, and HTNPs-1 displayed the higher current density and PCE values.

**Figure 7:**
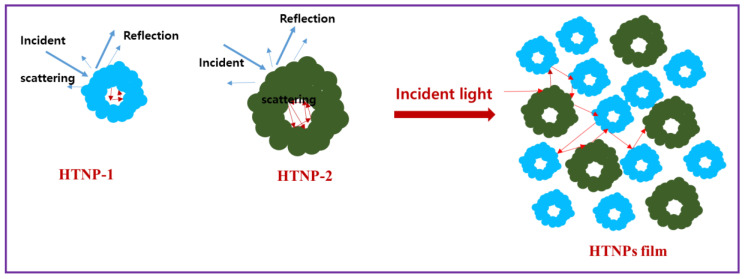
Schematic understanding of the light-scattering properties of hollow TiO_2_ nanostructured particle-based films for photovoltaic devices.

Given this outcome, the enhanced photoconversion efficiency of HTNPs photoanodes is attributed to the strong light scattering by the hollow interiors, increased dye adsorption and strong optical confinement in the HTNPs (Figure 7). The strong light-scattering properties of the C-TiO_2_ and H-TiO_2_ were confirmed by recording the UV-vis spectra, and the relevant spectra are provided in Figure S2. Both commercial TiO_2_ NPs and hollow TiO_2_ had high reflectivity in the wavelength range of 400–800 nm, which in turn improved the photoconversion efficiency. Further, the EPD films offered passage channels to infiltrate the electrolyte and dye molecules. Therefore, the photoelectrode film is made of hollow spheres that consist of nanocrystallite aggregates, and the aggregates also serve as scatterers along with the hollow interiors causing light scattering within the photoelectrode film. Such light scattering may be advantageous for the devices by extending the light-traveling distance within the HTNPs film and thus enhancing the probability of photons to interact with the dye molecules adsorbed on the surface of oxide nanocrystallites.

## 4. Conclusions

In summary, hollow TiO_2_ nanoparticles (HTNPs) were successfully obtained by microwave hydrothermal reaction and selective Au etching in Au@TiO_2_ core–shell NPs with KCN solution. Due to the light-scattering effect, the large-sized HTNPs were chosen as photoanode films for DSSC applications. By using electrophoretic deposition, hollow TiO_2_ NPs with hollow interiors of 20–30 and 70–80 nm size were effectively deposited as bi-layers and tri-layers on the TiO_2_ compact layer. The amount of HTNPs loading in the EPD process was controlled by the deposition time. The DSSCs based on HTNPs-1 exhibited a photoconversion efficiency of 6.33%, which is 33% higher than the DSSC device without the HTNPs-1 light-scattering layer. Further, the photoconversion efficiency is greatly improved to 8.08% with the tri-layer film of HTNPs-2s. The boosted conversion efficiency of bi-layered and tri-layered DSSCs is attributed to the efficient light scattering and enhanced light-harvesting properties of hollow TiO_2_ nanostructures, leading to an increased photocurrent density in the photoanodes. These results indicated that the HTNPs provide a promising choice of light-scattering layer for high stability and high-efficiency photovoltaic cells. Therefore, light-harvesting properties, electrolyte diffusion and electron transport are critical for the future preparation of high-efficiency DSSCs with hollow TiO_2_ spheres in the photoanodes.

## Figures and Tables

**Table 1:** Characteristic parameters of DSSCs made from compact TiO_2_ film and bi-layered and tri-layered films with different light-scattering layers.

Cell Type	V_oc_ (V)	Jsc (mA/cm^2^)	Fill Factor (FF in %)	Efficiency (%)
DSSC-1	0.59 (±0.05)	12.34 (±0.02)	65.27 (±0.1)	4.75 (±0.2)
DSSC-2	0.63 (±0.05)	15 (±0.02)	66.77 (±0.1)	6.33 (±0.2)
DSSC-35	0.67 (±0.05)	14.78 (±0.02)	69.5 (±0.1)	6.90 (±0.2)
DSSC-310	0.63 (±0.05)	17.2 (±0.02)	69.29 (±0.1)	7.58 (±0.2)
DSSC-315	0.68 (±0.05)	16.26 (±0.02)	72.33 (±0.1)	8.08 (±0.2)

## Data Availability

Data are available on request from the authors.

## Supplementary Materials

The following supporting information can be downloaded at: https://www.mdpi.com/article/10.3390/nano14221782/s1, Figure S1. FE-SEM cross-sectional image of bilayer film in which HTNP-1 were deposited on the C-TiO_2_ film via electrophoretic deposition. Figure S2. UV-vis reflectance spectra of C-TiO_2_ and Hollow TiO_2_ NPs.
