# Supplementary material for: Layer-by-Layer Deposition of Hollow TiO2 Spheres with Enhanced Photoelectric Conversion Efficiency for Dye-Sensitized Solar Cell Applications
Source: Nanomaterials (Basel). 2024 Nov 6;14(22):1782. doi: 10.3390/nano14221782 (PMC11597829; doi:10.3390/nano14221782)
Supplement: Supplementary file 1 [file nanomaterials-14-01782-s001.zip › nanomaterials-3262085-supplementary.pdf]

Layer-by-Layer Deposition of Hollow TiO_2 Spheres with Enhanced Photoelectric Conversion Efficiency for Dye-Sensitized Solar Cell Applications

Rama Krishna Chava[1], Yeon-Tae Yu[2],* and Misook Kang[1,*]

[1]Department of Chemistry, College of Natural Sciences, Yeungnam University, 280 Daehak-Ro, Gyeongsan, 38541, Republic of Korea; drcrkphysics@hotmail.com or rama@ynu.ac.kr

[2]Division of Advanced Materials Engineering, Jeonbuk National University, 567, Baekje-daero, Deokjin-gu, Jeonju, 54896, Republic of Korea.

*Correspondence: yeontae@jbnu.ac.kr (Y. –T. Yu); mskang@ynu.ac.kr (M. Kang)

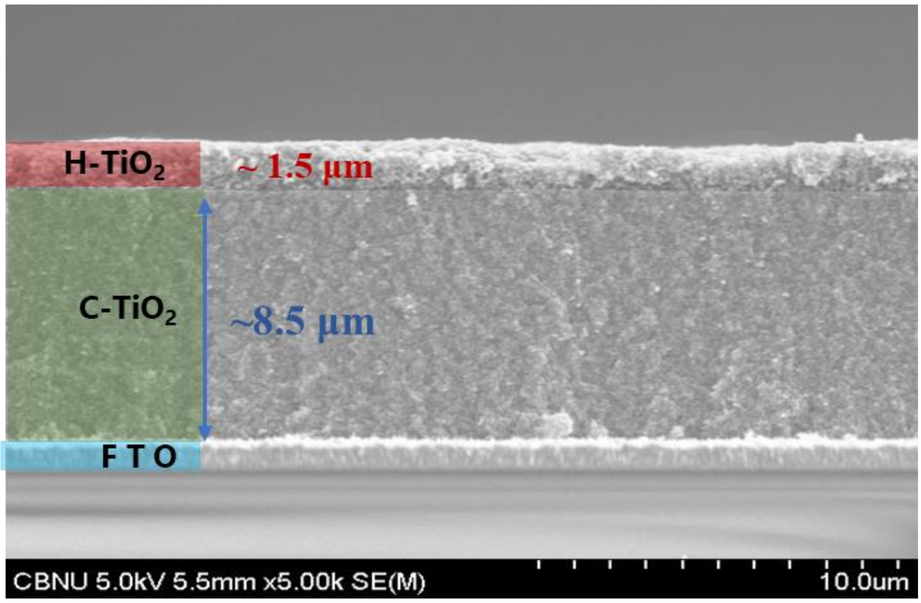

Figure S1. FE-SEM cross-sectional image of bilayer film in which HTNP-1 were deposited on the $C\text{-}TiO_2$ film via electrophoretic deposition.

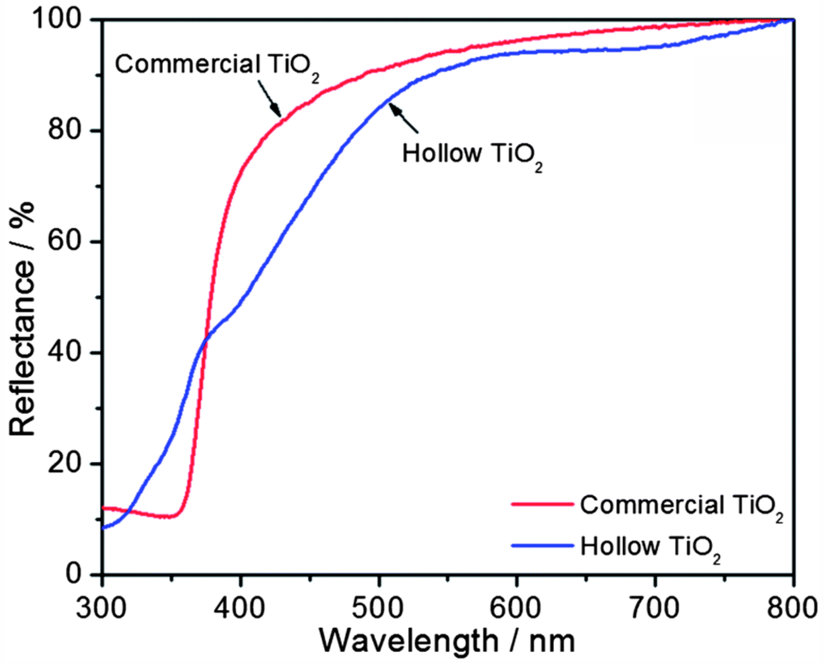

Figure S2. UV-vis reflectance spectra of $C\text{-}TiO_2$ and Hollow TiO_2 NPs
